# Toward structuring real-world data: Deep learning for extracting oncology information from clinical text with patient-level supervision

**DOI:** 10.1016/j.patter.2023.100726

**Published:** 2023-04-14

**Authors:** Sam Preston, Mu Wei, Rajesh Rao, Robert Tinn, Naoto Usuyama, Michael Lucas, Yu Gu, Roshanthi Weerasinghe, Soohee Lee, Brian Piening, Paul Tittel, Naveen Valluri, Tristan Naumann, Carlo Bifulco, Hoifung Poon

**Affiliations:** 1 Microsoft Research, Redmond, WA, USA; 2 Providence St Joseph’s Health, Portland, OR, USA; 3 Providence Genomics & Earle A. Chiles Research Institute, Portland, OR, USA

**Keywords:** natural language processing, L01.224.050.375.580, data mining, L01.313.500.750.280.199, medical oncology, H02.403.429.515, neoplasm staging, E01.789.625

## Abstract

Most detailed patient information in real-world data (RWD) is only consistently available in free-text clinical documents. Manual curation is expensive and time consuming. Developing natural language processing (NLP) methods for structuring RWD is thus essential for scaling real-world evidence generation. We propose leveraging patient-level supervision from medical registries, which are often readily available and capture key patient information, for general RWD applications. We conduct an extensive study on 135,107 patients from the cancer registry of a large integrated delivery network (IDN) comprising healthcare systems in five western US states. Our deep-learning methods attain test area under the receiver operating characteristic curve (AUROC) values of 94%–99% for key tumor attributes and comparable performance on held-out data from separate health systems and states. Ablation results demonstrate the superiority of these advanced deep-learning methods. Error analysis shows that our NLP system sometimes even corrects errors in registrar labels.

## Highlights

• Patient-level supervision supercharges learning to structure real-world data (RWD) at scale
• Deep-learning methods extract key oncology attributes with high performance (AUROC: 94%–99%)
• NLP system may sometimes correct errors in medical registrar labels
• Extensive study from cancer registry of large integrated development network (IDN)

## The bigger picture

The use of real-world data (RWD) in healthcare has become increasingly important for evidence generation. However, much detailed patient information is only consistently available in free-text clinical documents, and manual curation is expensive and time consuming. This paper proposes using information in medical registries, which are often readily available and capture patient information, as the basis for patient-level supervision to train deep-learning systems that facilitate general RWD applications. This proposed approach has potential to make evidence generation scalable and efficient.

An extensive study on 135,107 patients from the cancer registry of a large integrated delivery network (IDN) comprising healthcare systems in five western US states demonstrates the effectiveness of this approach. The resulting deep-learning models demonstrate high performance in extracting key tumor attributes, and error analysis shows that they can even correct errors in registrar labels.

## Teaser

The use of real-world data (RWD) in healthcare has become increasingly important for evidence generation. However, much detailed patient information is only consistently available in free-text clinical documents, and manual curation is expensive and time consuming. This article proposes using information in medical registries, which are often readily available and capture patient information, as the basis for patient-level supervision to train deep-learning systems that facilitate general RWD applications. This proposed approach has the potential to make evidence generation scalable and efficient.

## Introduction

Electronic medical records (EMRs) offer an unprecedented opportunity to harness real-world data (RWD) for accelerating progress in clinical research and care.^1^ By tracking longitudinal patient care patterns and trajectories, including diagnoses, treatments, and clinical outcomes, we can help assess drug efficacy in real-world settings, facilitate post-market surveillance, and speed up clinical trial recruitment. However, pertinent information about patients often resides in clinical text, such as pathology assessments, radiology assessments, and clinical progress notes. Manual curation to structure such text is expensive and hard to scale.

Natural language processing (NLP) can help accelerate manual curation.^2^ In recent years, there have been rapid advances in general-domain NLP, where state-of-the-art deep neural networks, such as transformer-based models including bidirectional encoder representations from transformers (BERT), have demonstrated remarkable success across a wide range of applications.^3^^,^^4^ Training these sophisticated models, however, typically requires a large number of annotated examples. By contrast, prior work in clinical NLP is often limited to annotating small datasets and training simpler methods.^5^ Due to the scarcity of qualified domain experts, annotation is usually conducted on a small collection of notes, often from a single institution. Moreover, to make learning easier, these explorations typically restrict annotation to single sentences or single notes. For example, Kehl et al.^5^ show promising results for applying NLP to accelerate real-world evidence generation in oncology. However, while their annotation effort is relatively large among similar prior efforts, their test set contains only 109 patients (1,112 patients in the entire annotated dataset). The notes are limited to radiology reports for lung cancer from a single institution. Their exploration is limited to convolutional neural networks, which do not leverage the latest NLP advances, such as language model pretraining.^6^^,^^7^

In this article, we propose to bootstrap deep learning for structuring RWD by using readily available registry data. Medical registries are routinely collected for various diseases, with oncology being a prominent example. In the US, cancer is a reportable disease, and cancer centers are required to curate patient information per national accreditation and clinical quality requirements. By matching registry entries with their corresponding EMR data, we can assemble a large dataset for training and evaluating state-of-the-art deep NLP methods.

Gao et al.^8^^,^^9^ also leverage registry data for supervision. However, like Kehl et al.,^5^ they restrict classification to individual pathology reports and exclude tumors associated with multiple reports. Similarly, Percha et al.^10^ focus on classifying individual sentences for breast cancer surgery information. Such methods are not applicable to the prevalent cases where information is scattered across multiple clinical documents and note types (e.g., pathology reports, radiology reports, progress notes). Often, information in a single document (e.g., discussion of a malignant site) is insufficient, and additional context is required for identifying the correct diagnosis or staging information.

To the best of our knowledge, our study is the first to explore cross-document medical information extraction using registry-derived, patient-level supervision to train deep NLP methods. Such patient-level supervision is inherently challenging to use as it comprises only annotations associated with a tumor diagnosis, which are not attributable to individual sentences or documents. Each patient may have dozens of clinical documents, yielding very long input text spans that are difficult to process for standard deep-learning methods. Additionally, the collection of clinical documents spans decades and varies in completeness. Nevertheless, we found that the scale of such self-supervised data more than compensates for their noise and technical challenges, and our models attain high performance (area under the receiver operating characteristic curve [AUROC]: 94%–99%) for extracting core tumor attributes such as site, histology, and clinical/pathological staging.

Unlike settings of prior studies,^8^ sophisticated deep-learning methods substantially outperform simplistic approaches, with our top-performing model combining cutting-edge techniques such as transformers,^3^ domain-specific pretraining,^7^ recurrent neural networks,^11^ and hierarchical attention.^12^ Our method naturally handles longitudinal information, and experiments show that incorporating multiple document types significantly improves performance. Neural attention can be used to pinpoint relevant text spans as extraction rationale and provenance, which facilitate model interpretation and rapid validation by human experts. Our model, trained on a health system in one state, performed comparably for patients from different states, health systems, and EMR configurations, suggesting good generalizability.

While our work is motivated by structuring RWD, our method can also be used to accelerate registry curation. Our deep learning model not only performs well in abstraction but also attains high accuracy in case finding (identifying patients for cancer registry), thus paving the way for end-to-end-assisted cancer registry curation.

## Results

We conduct experiments using data from a large integrated delivery network (IDN) with over 28 distinct cancer care centers across US states. We assemble a dataset with patient-level supervision by matching comprehensive EMR records (including all free-text clinical documents in scope here) and cancer registry records. Patients without a digitized pathology report within 30 days of diagnosis are skipped. This yields a total of 135,107 patients spanning multiple US states between 2000 and 2020. We use patients in Oregon for the initial exploration (n=39,064, 29% of patients). We divide patients into ten random folds. We use six folds for training and development (n=23,438), two folds for test (n=7,745), and two folds for an additional held-out test set (n=7,881). We reserve patients from Washington (n=36,900), as well as the remaining states (n=59,143) for further generalizability tests, with a distinct health system being used in each state.

Medical abstraction can be formulated as a binary classification problem: given clinical text *T* for a patient, attribute *A*, and a particular value *a*, classify if *A*’s value as described in *T* is *a* (*a* can be null if *T* contains no mention of *A*). In this article, we focus on three types of core cancer attributes: tumor site, histology, and staging. In each patient instance, the input comprises pathology report, radiology reports, and operative notes, concatenated chronologically.

We use the ICD-O-3 ontology for tumor site and histology. For staging, we focus on solid tumors and follow AJCC guidelines for clinical and pathological staging. Both represent cancer progression using TNM classification (T is tumor size/location, N is lymph node status, and M is metastasis). Clinical staging is based on initial diagnosis using medical imaging, clinical assessment, and/or biopsy, whereas pathological staging incorporates more definitive assessments of the tumor size and spread. For simplicity and based on practical utility, we focus on classifying coarse categories (T: 0–4, *in situ*; N: 0 vs. 1+; M: 0 vs. 1).

For each attribute, we report the standard AUROC. For system comparison, however, AUROC might obscure key performance differences in the presence of imbalanced distribution (e.g., some sites appear much more frequently), so we evaluate area under the precision-recall curve (AUPRC). Precision and recall are also known as positive predictive value and sensitivity, respectively. We also report accuracy for completeness. In all cases, we report micro scores aggregated across all classes.

### Deep learning effectively extracts key oncology attributes

Table 1 shows test results for extracting key oncology attributes. By incorporating state-of-the-art advances such as PubMedBERT and OncoBERT, our deep-learning system attains high performance across the board, even for tumor site and histology, where the system has to distinguish among hundreds of labels. Despite the large parameter space, our system is robust in experiments, with standard deviations across two random runs smaller than 1% for all tasks.

**Table 1:** Test results for oncology abstraction by our deep learning system based on PubMedBERT (PubMed) and OncoBERT (Onco)

	AUPRC		AUROC		Accuracy	
	PubMed	Onco	PubMed	Onco	PubMed	Onco
Tumor site	76.7	77.1	99.3	99.2	69.1	69.5
Histology	87.2	87.6	99.4	99.4	81.2	81.2
Clinical T	79.3	81.4	93.9	94.6	70.1	72.0
Clinical N	97.2	97.5	97.2	97.5	91.6	92.3
Clinical M	98.7	99.0	98.7	99.0	94.9	95.2
Pathologic T	87.2	87.6	96.1	96.1	78.6	79.1
Pathologic N	95.3	95.5	95.2	95.4	88.9	88.8
Pathologic M	98.6	98.9	98.6	98.9	95.1	95.6

The ICD-O-3 ontology is used for tumor site and histology. Clinical and pathological staging use TNM classification (T is tumor size/location; N is lymph node status; M is metastasis).

### Generalizability

To assess generalizability, we evaluate the held-out set and find that model performance is nearly identical. We further evaluate our model, trained on the Oregon training set, on patients from Washington and other states. Each state has a distinct cancer registry system, operated independently and governed by state laws. Therefore, held-out states offer a particularly good test for generalizability (see Table 2). The results are comparable for most attributes, with only slight degradation. Histology, however, shows a large performance decrease (87.2 vs. 80.5 and 78). Manual analysis shows that much of this drop is attributable to differences in curation standards, with registrars from different systems using different labeling granularity, e.g., non-small cell lung cancer (8,046) vs. adenocarcinoma (8,140), with the latter being the most common type of the former. Clinical tumor (T) staging also shows a noticeable performance decrease (79.3 vs. 73.5). Manual analysis shows that this performance drop largely stems from a higher proportion of highly ambiguous cases (e.g., borderline categories between stages 2 and 3).

**Table 2:** Generalizability test (AUPRC) on Oregon (OR), Washington (WA), and other states using our deep learning models (based on PubMedBERT) trained on Oregon training

	OR test	OR held out	WA	Other states
Tumor site	76.7	76.4	73.5	73.0
Histology	87.2	87.6	80.5	78.0
Clinical T	79.3	78.8	73.5	73.5
Clinical N	97.2	97.6	95.4	96.0
Clinical M	98.7	98.8	97.3	97.7
Pathologic T	87.2	88.0	84.3	86.1
Pathologic N	95.3	95.7	92.9	95.1
Pathologic M	98.6	98.6	97.1	97.1

Washington (WA) and other states all use different health systems. There is only slight degradation for most results, which bodes well for generalizability of our models. A notable exception is histology, with up to a nine-point drop. Upon close inspection, this stems from divergence in curation standards on ambiguous cases, with registrars using different labeling granularity (e.g., non-small cell lung cancer vs. lung adenocarcinoma).

### System comparison

Table 3 compares our deep-learning systems with prior approaches for medical abstraction. An ontology-aware rule-based system (matching against class lexicon and known aliases) performs poorly, demonstrating that entity recognition alone is inadequate for such challenging tasks. Deep-learning methods perform substantially better, with BERT-based approaches outperforming convolutional neural networks (CNNs), especially for the most challenging tasks such as site, histology, and clinical/pathological T staging. Hierarchical attention network (HAN)/gated recurrent unit (GRU) and transformer-based language models each contribute significantly, with our best system gaining 5.1 points for site, 3.2 points for histology, and 7.2 points for clinical T over GloVe+CNN.

**Table 3:** Comparison of test AUPRC scores for oncology abstraction by various NLP systems

	Site	Histology	Clin. T	N	M	Path. T	N	M
Ontology	19.4	19.2	–	–	–	–	–	–
BOW	62.8	76.6	70.4	96.6	98.4	72.1	90.7	98.9
OncoGloVe+CNN	72.0	84.4	74.2	96.5	98.6	83.9	93.1	98.5
OncoGloVe+HAN/GRU	74.0	85.9	76.2	97.1	98.7	86.4	94.2	98.5
BERT+HAN/GRU	75.1	86.2	77.0	96.6	98.4	86.4	94.4	98.2
PubMedBERT+HAN/GRU (ours)	76.7	87.2	79.3	97.2	98.7	87.2	95.2	98.6
OncoBERT+HAN/GRU (ours)	77.1∗	87.6∗	81.4∗	97.5∗	99.0∗	87.6∗	95.5∗	98.9∗

Ontology, ontology-aware rule-based system; BOW, logistic regression with bag-of-word features; OncoGloVe, 100-dimensional GloVe embedding pretrained on oncology notes.

∗ Highest performance for each abstraction task (column).

Domain-specific pretraining is especially impactful. By pretraining entirely on oncology notes, OncoBERT further improves over PubMedBERT, which is already pretrained on biomedical text. Compared with general-domain BERT, our best system with OncoBERT gains 2.0 points for site and 4.4 points for clinical T staging.

### Ablation study

We incorporate three types of clinical documents as input: pathology reports, radiology reports, and operative notes. In ablation study, we find that having all three helps, presumably because this increases robustness in case some relevant notes are missing or not yet digitized (e.g., scanned PDFs). In other words, adding radiology reports on top of pathology reports increased the AUPRC by 3.4 absolute points for tumor site extraction, with the inclusion of operative notes providing an additional one-point gain. By default, we use [−30, 30] days around diagnosis, which works reasonably well in general. For pathological staging, however, a larger window is helpful, as relevant information often comes from a tumor surgical resection that may be several months after an initial tissue biopsy or fine-needle aspiration-based diagnosis, e.g., using [−30, 90] days as input improves the AUPRC by over four absolute points for pathological T staging (87.2 vs. 91.8).

### Case finding

In medical abstraction, we are given patients with cancer and asked to extract key tumor attributes. By contrast, the goal of case finding is to determine if a patient should be included for cancer registry. Cancer providers are obligated to submit abstraction for these patients to the registry within a time limit. Therefore, it’s important to identify such cases as soon as possible and to start the abstraction process. We assemble a case-finding dataset using patients in the cancer registry. For positive cases, we identify patients with cancer with at least a pathology report on the day of diagnosis. For negative cases, we randomly sample non-cancer patients. This yields 62,090 positive and 8,460 negative patients. We divide them into train/development/test by 60%/20%/20%, with 12,418 positive and 1,692 negative patients in the test set.

A patient may have clinical documents on multiple days. In case finding, a classification instance comprises a patient’s clinical documents in a given day, and the ultimate goal is to identify the moment of cancer diagnosis (when registry curation starts). For evaluation, we adopt a patient-level metric that mirrors real-world applications. For each patient, we return the first day with positive classification. For patients with cancer, the case-finding decision is deemed correct if the first day of positive classification is within [−7, 30] days of diagnosis. For non-cancer patients, the case-finding decision is deemed correct if all classifications are negative. The [−7,30] window is chosen based on consultations with subject-matter experts, as information about cancer diagnosis may not be recorded exactly on the diagnosis date. We report the F1 score, which is the harmonic mean of precision (positive predictive value) and recall (sensitivity). Specifically, F1=2/(1/precision+1/recall).

For self-supervision, we explore the two settings as described in the experimental procedures. In both cases, positive instances comprise patients with cancer on the diagnosis date. By default, negative instances comprise of randomly chosen days among non-cancer patients. Additionally, we randomly sample days at least a week before diagnosis (up to a year before) among patients with cancer, subject to the condition that clinical documentations are available on the given days. This yields 9,836 instances as hard negative examples to add to the training set. With the base setting, we attain a test F1 score of 91.4. As shown in Table 4, by incorporating hard negative examples, we substantially improve the test F1 score to 97.3, gaining six absolute points.

**Table 4:** Comparison of test results in case finding with two self-supervision schemes

Self-supervision	Train positive instances	Train negative instances	Test F1
Default	37,207	13,123	91.4
+ Hard negatives	37,207	22,959	97.3

## Discussion

### Error analysis

We conduct manual analysis on sample errors. Some stem from annotation inconsistency, where registrars actually agree with our system classifications upon close inspection. Others stem from missing notes. After adjusting for annotation inconsistency and missing input, the real test performance of our deep-learning system is even higher. For example, by analyzing 50 error examples for tumor site classification, we found that a significant proportion of them stemmed from incorrect annotations, based on which we could estimate that the real test AUPRC is about 91.6 (vs. 76.7).

### Assisted curation

**Figure 1:**
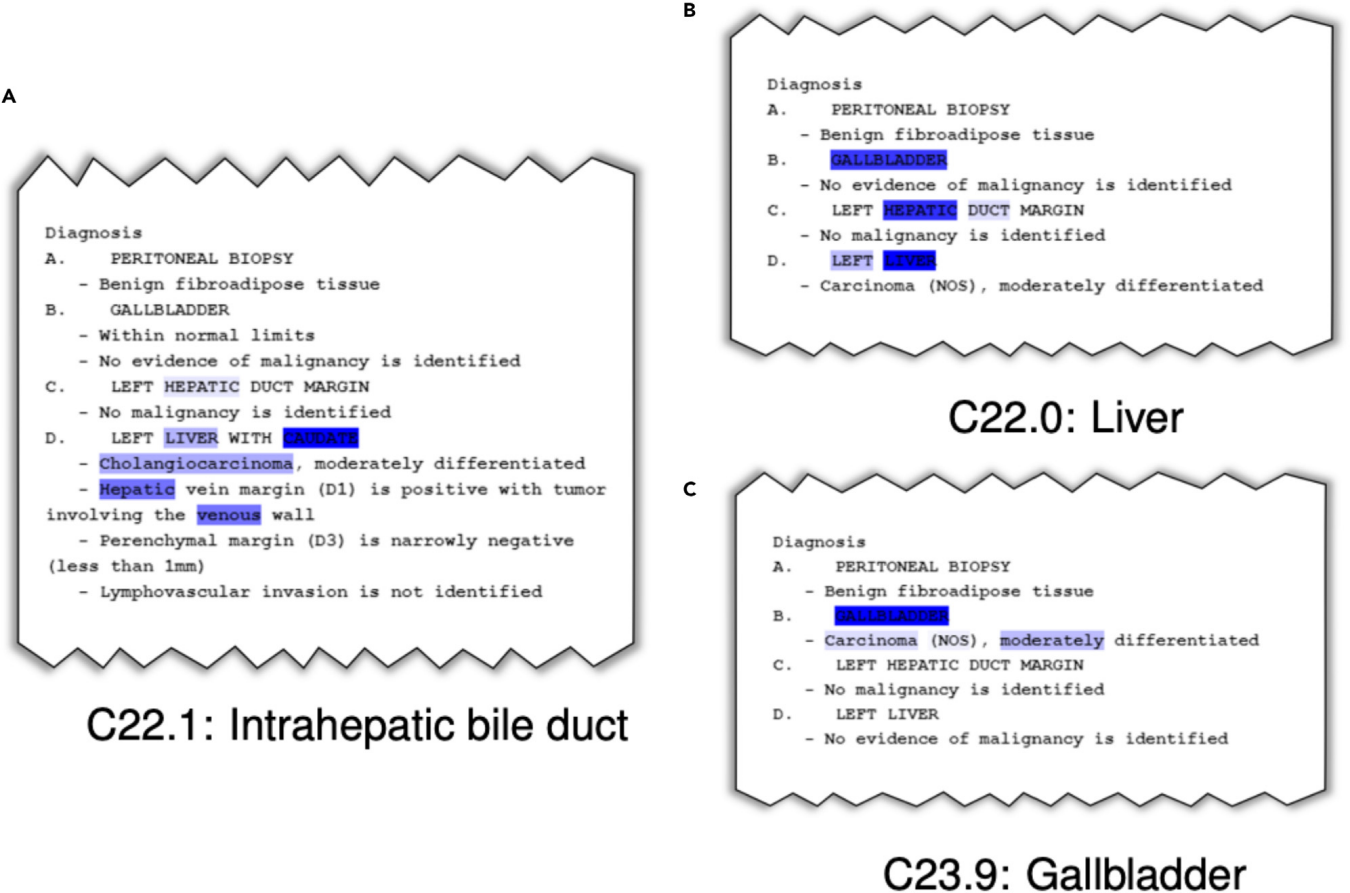
Examples of observed attention patterns and predictions from the tumor site model (A) The attention pattern for the example shown in Figure 2A, with darker color signifying higher attention weight. The tumor site model correctly identifies C22.1 (intrahepatic bile duct) due to the cholangiocarinoma histology (indicating cancer of the bile duct). To probe the model understanding further, inference was run on modified text. (B) The description was changed to a generic “carcinoma” diagnosis. While the attention is more diffuse, the model places the highest attention on the “liver” section and correctly identifies C22.0 (liver) as the tumor site. (C) The “carcinoma” diagnosis was moved to the “gallbladder” section, and the model now correctly identifies the site as C23.9 (gallbladder), with attention now focusing on this section.

**Figure 2:**
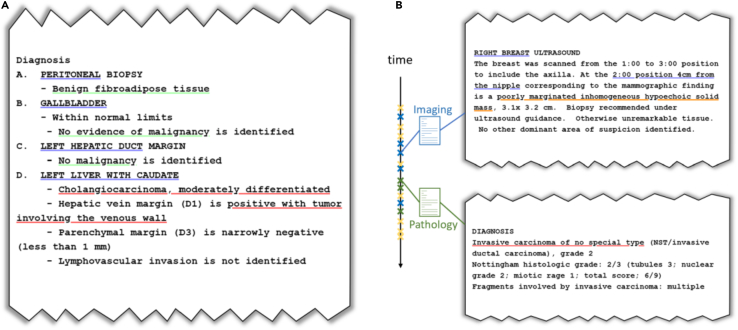
An example of semantics tumor site extraction Simple NLP methods are not sufficient to handle complex semantics in general medical abstraction, as can be seen in the example of tumor site extraction. (A) Named entity recognition (NER) is not enough; many candidate sites may be present, but the correct tumor site must be associated with a positive diagnosis. (B) Abstraction may require cross-document extraction; in this example, the location is described in an imaging report, whereas the positive diagnosis is documented in a pathology report. Our sophisticated transformer-based model can classify them correctly and identify relevant rationale via attention. In these examples, blue underlining shows body sites, and green, orange, and red underlining show indications of negative, possible, and positive cancer diagnoses, respectively.

**Figure 3:**
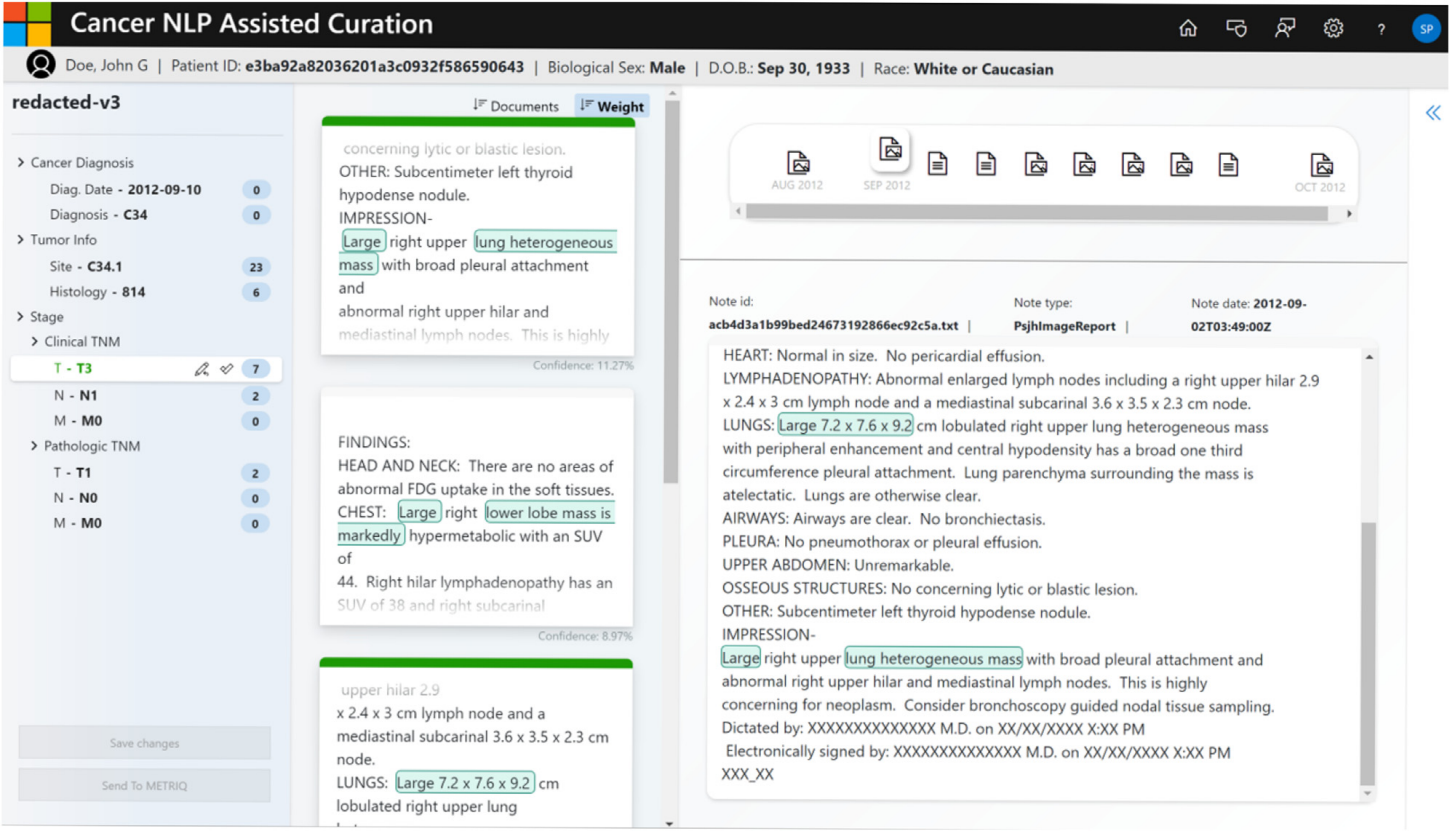
Cancer NLP-assisted curation system Our cancer-assisted curation system. Left: extracted oncology attributes. Middle: extraction rationale based on attention weights. Right: full notes. Patient information has been deidentified.

We envision that NLP extraction can serve as a candidate to help accelerate curation. The attention mechanism in transformer-based models provides a straightforward approach to identify extraction rationale. Effectively, the aggregate representation of the input text is the weighted sum of token representations in the top layer, with the weights (derived from self-attention to the special [CLS] token) signifying relative importance of individual tokens in the final classification decision. While there is no guarantee that attention provides explanation,^13^ in practice, we find that tokens with the highest attention weights are conducive to assisted curation and generally conform with what human experts would consider as extraction rationale. As an example, Figure 1 highlights tokens with high attention weights for the example text in Figure 2A and two variations. While the attention may not entirely align with individual human intuition, it broadly conforms to the extraction rationale and enables quick verification. Figure 3 shows a research prototype that we have developed for assisted curation, which is in test use by selected clinical users. For each attribute, the interface displays the extraction rationale by highlighting individual notes and text spans with the highest neural attention weight for final classification. In preliminary studies, tumor registrars can verify a candidate extraction in 1–2 min, either ascertaining its correctness or fixing the label in the interface. A thorough evaluation of this system is out of the scope for this article, and we leave it to future work.

### Fairness

To assess fairness in our models over subpopulations, we conducted a performance evaluation for each gender and ethnicity subgroup in the test set (two folds of Oregon patients). Note that the gender and ethnicity information was never used by any of our models during training. Following disparate impact metric (80% rule),^14^ equal performance was observed on almost all scenarios, except for tumor site abstraction on the subgroup “Native Hawaiian or Other Pacific Islander.” Using accuracy as the evaluation metric, model performance for tumor site abstraction is 69% of that for the subgroup “White or Caucasian” (48.3 vs. 69.6). We next investigate if this instance of underperformance stems from any bias in our training process. In particular, even though ethnicity is not explicitly specified during training, relevant information may be present in the notes and discerned by the model. Similarly, ethnic stereotypes and biases may be reflected in pretrained embeddings.^15^^,^^16^ To test if these might have contributed to the above case of underperformance, we applied a standard protected health information (PHI) extraction model to extract ethnicity-revealing tokens such as geography or surname. We then identified tokens receiving top attention from the tumor site abstraction model and compared the two sets. We found that less than 2% of top-attention tokens were ethnicity-related tokens, which means that it is unlikely that the tumor site abstraction model overfitted to ethnicity information, resulting in modeling bias.

Instead, we conclude that the most likely explanation is random fluctuation stemming from the very small sample size of the test set for this subgroup (only 29 patients). Specifically, we conducted additional evaluations using all reserved data from other locations. This resulted in over an order of magnitude more data across all subgroups, and we observed less than 3% difference between “Native Hawaiian or Other Pacific Islander” and other subgroups. This suggested that the performance gap we observed in the original test set likely stemmed from statistical noise due to the small sample size for this subgroup other than fundamental bias in modeling and training.

### Limitations of the study

Our study focuses on medical abstraction of key diagnosis information as curated in cancer registry. Future work should explore extraction of treatment and outcome information, as well as other diagnostic information such as biomarkers. Cancer registries focus on complete curation of “analytical cases,” i.e., patients with both initial diagnosis and treatment occurring within a given healthcare system. The models may perform less well for patients who are initially diagnosed elsewhere and then referred to the given network, e.g., due to missing digitized reports. In many such cases, PDFs or scanned documents are still available. We are exploring the use of state-of-the-art document image understanding methods, such as LayoutLM,^17^ with initial promising results. Our immediate exploration of assisted curation focuses on accelerating case identification and medical abstraction, but it also opens up opportunities for interactive learning to continuously improve machine reading based on user feedback. In addition to improving abstraction accuracy, this can potentially help calibrate attention weights for extraction rationale.^18^ Pretraining can also be further improved by incorporating domain knowledge, such as from the Unified Medical Language System (UMLS).^19^^,^^20^

### Toward scaling RWD curation

Manual curation of complex clinical records and EMR data is expensive and time consuming. The healthcare network represented in this study hires several dozen full-time registrars for cancer registry abstraction. Curation is limited to analytic cases (i.e., those first treated in a given cancer center), which are required for reporting, thus skipping a large swath of patients. Despite such restrictions and significant investment, there is still significant delay for a majority of the patients. To estimate the extent of curation backlog, we analyze two snapshots of a cancer registry that are 8 months apart. Among newly curated cases in the second snapshot, 23,670 are diagnosed before the first snapshot ends. They have a median of 324 days between diagnosis and the first snapshot end date. Many cases are curated over a year after diagnosis. By leveraging assisted curation with candidate abstractions generated by our deep NLP system, we can accelerate cancer registry abstraction and reduce backlog. Given promising results in the preliminary study, we are now exploring integration of assisted curation to the registry abstraction workflow.

NLP-based machine reading also helps scale RWD curation. The healthcare network in our study has over 1.2 million patients with cancer with digitized pathology reports within 30 days of diagnosis. However, only 135,107 of them have been curated in the cancer registry. By applying our NLP system to all patients, we instantly expand structured RWD for the network by an order of magnitude. In future work, we plan to expand the scope of curation by applying self-supervised learning to extracting other key information for real-world evidence, such as treatments and key clinical outcomes.^21^^,^^22^^,^^23^^,^^24^ A particularly exciting research frontier lies in studying response to immunotherapy, such as check-point inhibitors (CPIs). In preliminary study, we find that self-supervised NLP methods can immediately identify and abstract over an order of magnitude more patients treated with CPIs compared with prior manual efforts that took many months.

## Experimental procedures

### Resource availability

#### Lead contact

Further information and requests for resources should be directed to the corresponding authors, Carlo Bifulco (carlo.bifulco@providence.org) and Hoifung Poon (hoifung@microsoft.com). All other queries can be directed to the lead contact, Hoifung Poon (hoifung@microsoft.com).

#### Materials availability

This study did not generate any physical materials.

### Human subjects/IRB, data security, and patient privacy

This work was performed under the auspices of an institutional review board (IRB)-approved research protocol (Providence protocol ID 2019000204) and was conducted in compliance with human subjects research and clinical data management procedures—as well as cloud information security policies and controls—administered within Providence St. Joseph Health. All study data were integrated, managed, and analyzed exclusively and solely on Providence-managed cloud infrastructure. All study personnel completed and were credentialed in training modules covering human subjects research, use of clinical data in research, and appropriate use of IT resources and IRB-approved data assets.

### Methods

#### Abstraction

**Figure 4:**
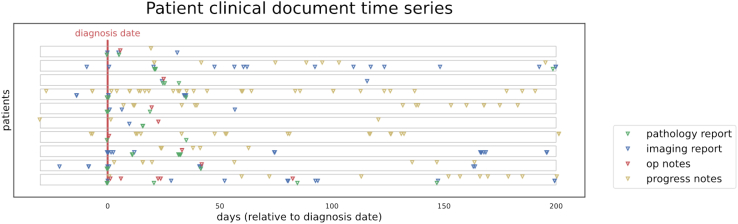
Patient clinical document time series Patients with cancer typically have many clinical documents for a tumor diagnosis, with key information scattered among these documents.

Medical abstraction can be formulated as information extraction in NLP. Given clinical text *T* for a patient and attribute *A*, the goal is to extract *A*’s value as described in *T* (or the absence thereof), which can be framed as a binary classification problem (by enumerating all possible values *a*). In most prior work, *T* is a sentence or a clinical note, and *A*’s value only has a few choices (e.g., the presence or absence of active cancer^5^). By contrast, we consider the most general setting, where *T* comprises all notes for a patient and *A*’s range may number in the hundreds, e.g., there are 310 classes for tumor site and 556 for histology in ICD-O-3, and a patient may have many notes (Figure 4).

**Figure 5:**
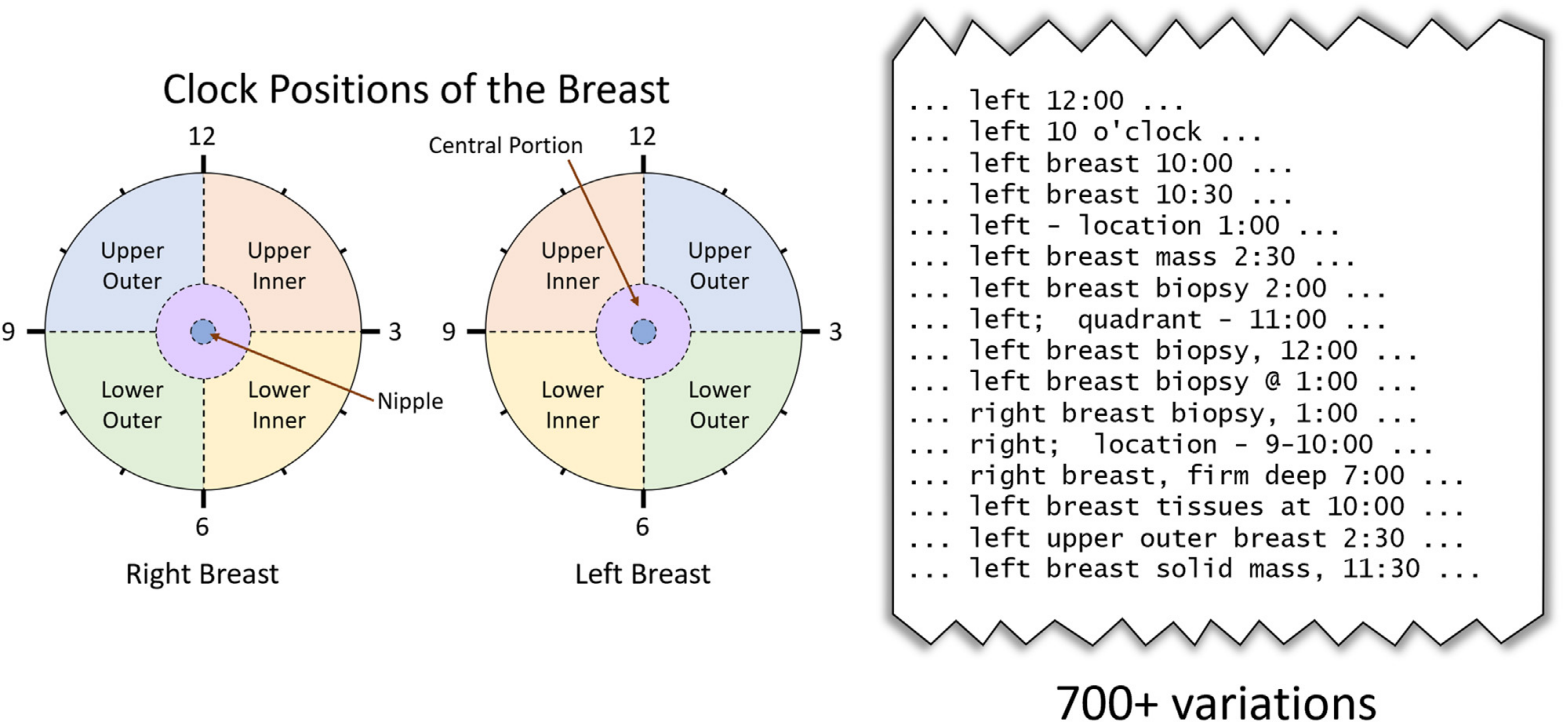
Variations in medical abstraction Relevant information for medical abstraction may manifest in myriad variations, as seen in specification of tumor site in breast cancer with laterality and clockwise position.

In general, abstraction presents substantial challenges for NLP systems. Relevant information may manifest in myriad variations (Figure 5). Named-entity recognition (NER) is not enough, as abstraction is more about extracting underlying relations, e.g., abstracting a tumor site is not about recognizing site mentions but is about determining if the patient has malignancy at the given site on a given date (Figure 2A). Moreover, abstraction may require information integration across multiple clinical documents (Figure 2B).

With patient-level supervision from medical registries, our machine-learning setting can be regarded as a form of distant supervision or, more generally, as self-supervision,^26^ as the labels cannot be attributed to a sentence or even a clinical document. However, given the aforementioned complex linguistic phenomena in medical abstraction, we do not generate noisy training examples by associating a label with a specific text span (e.g., individual sentences with the presence of relevant entities), as in standard distant supervision. Instead, we combine all clinical documents for a patient as input and rely on the deep-learning method to automatically identify pertinent sentences and notes.

#### Related work

Traditional clinical NLP systems are often rule based, e.g., leveraging regular expressions and domain lexicons from ontologies.^27^ They require significant efforts to build and may be vulnerable to linguistic variations and ambiguities. Consequently, machine-learning methods have seen increasing adoption.^28^ Traditional learning-based NLP methods require users to provide feature templates for classification, whereas modern deep-learning methods forgo this requirement and can automatically transform input text into a neural feature representation (a real-number vector).^5^^,^^6^^,^^8^^,^^9^^,^^29^

#### Deep learning for medical abstraction

**Figure 6:**
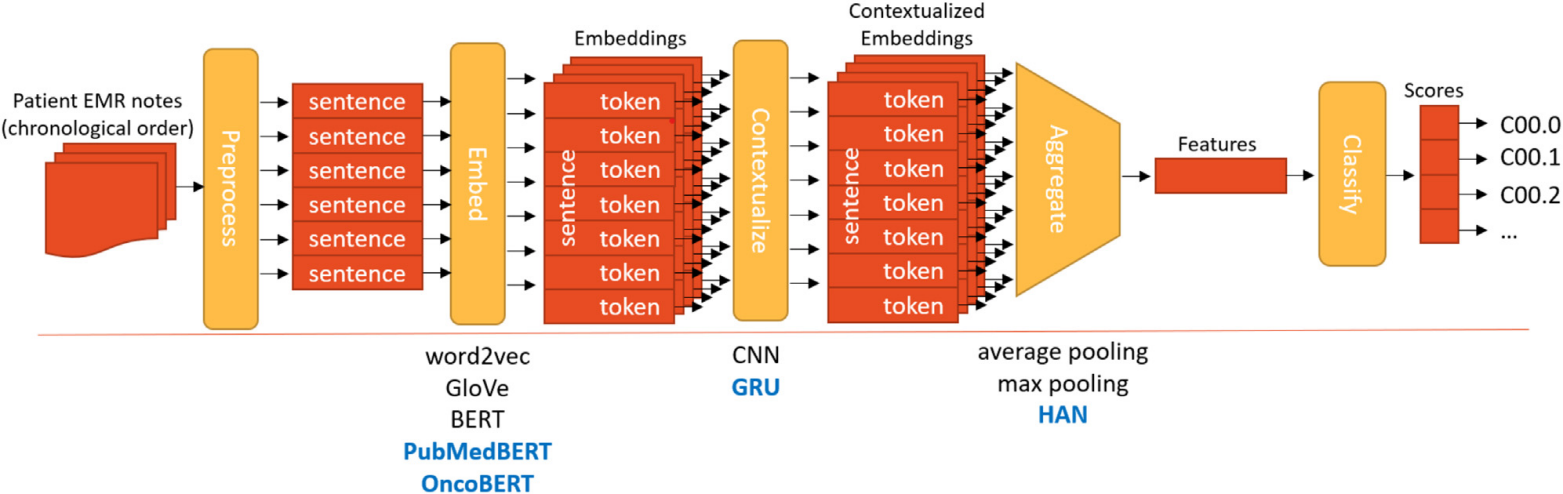
A general neural architecture for medical abstraction Clinical documents are concatenated by chronological order and converted into a token sequence, which is then transformed into a sequence of neural vectors by the embedding and contextualization modules, before being converted into a fixed-length feature vector by an aggregation module for final classification.

Figure 6 shows a general deep-learning architecture for medical abstraction. Medical documents are ordered temporally and converted into a sequence of sentences. They are tokenized and converted into a neural representation by an embedding module where each token is turned into a real-number vector. The vectors are then updated by a contextualization module and combined into a fixed-length feature vector by an aggregation module, which the classification module uses as input to produce the final classification.

In prior work applying deep learning to medical abstraction, the embedding module generally uses simple context-free embedding such as word2vec^30^ or GLoVE.^31^ Contextualization is usually done by CNN, which runs a sliding window over the tokens and generates output vectors using a shared neural network, with aggregation done by pooling.

Recently, there has been substantial progress in deep NLP methods. Transformer,^32^ with its multi-layer, multi-head self-attention mechanism, has proven very effective in modeling long-range dependencies and leveraging GPU parallelism. Contextualized embedding from language model pretraining^3^^,^^33^ is much more powerful than context-free embedding such as Word2Vec and GLoVe at extracting semantic information from unlabeled text and modeling variations/ambiguities. While the bulk of pretraining work focuses on general domains such as newswire and the Web, domain-specific pretraining has proven beneficial for specialized domains such as biomedicine by prioritizing learning of biomedical terms in relevant biomedical contexts.^7^^,^^34^^,^^35^

In this article, we conduct a thorough study of advanced deep NLP techniques in medical abstraction (Figure 6, blue). Some prior work investigated deep NLP in simplistic settings (e.g., classifying individual pathology reports) and concluded that advanced techniques such as transformer do not help their tasks.^8^ By contrast, we find that in the real-world setting of cross-document medical abstraction, advanced NLP techniques can confer significant benefit in combating the prevalent noises and linguistic complexities.

For embedding, we use the state-of-the-art biomedical neural language model PubMedBERT.^7^ The input to a neural language model consists of text spans, such as sentences, separated by special tokens [SEP]. To address the problem of out-of-vocabulary words, neural language models generate a vocabulary from subword units^36^^,^^37^ by greedily identifying a small set of subwords that can compactly form all words in a given corpus. BERT^3^ is a state-of-the-art language model based on transformer,^32^ which is pretrained by predicting held-out words in unlabeled text. While most BERT models were pretrained on general-domain text,^3^^,^^4^ PubMedBERT instead uses a biomedicine-specific vocabulary and was pretrained on biomedical literature from scratch. We also pretrained an oncology-specific OncoBERT on EMRs from over one million patients with cancer and explored its use in oncology abstraction. The pretraining was the same as in PubMedBERT^7^ except that the text comprises oncology notes rather than PubMed papers.

Self-attention requires pairwise computation among tokens, which scales quadratically in input text length. Consequently, standard BERT models typically limit input length (e.g., 512 tokens). This is not a problem for restricted settings such as sentence-level or document-level abstraction in prior work, but it poses a substantial challenge in the general setting, as patient-level, cross-document input has a median length of over 4,000 tokens. To handle such long text, we use GRU^11^ for contextualization and HAN^12^ for aggregation. GRU helps propagates information beyond BERT’s default length limit, and HAN provides better aggregation than pooling by weighing relevant tokens higher. The classification module is a standard linear layer followed by softmax, which produces multi-nomial probabilities among possible labels.

Our investigation differs in three important aspects. First, we consider a previously unexplored problem formulation. To the best of our knowledge, we are the first to explore cross-document medical abstraction, which poses significant challenges as mentioned in the article.

Second, standard deep-learning methods cannot handle long text spans as required in cross-document abstraction. We propose a novel combination of three cutting-edge deep-learning techniques for tackling these challenges in cross-document abstraction, as mentioned above and highlighted in blue in Figure 6. Specifically, we leverage a transformer-based, domain-specific foundation model (PubMedBERT or OncoBERT) to generate good sentence-level encoding, then use a recurrent neural network (GRU) to propagate information across sentences, and finally summarize information across multiple documents using HAN. As shown in ablation study (e.g., Table 3), this unique combination outperforms all prior deep-learning approaches, with all three components contributing significantly.

Finally, we propose to leverage patient-level labels readily available in cancer registry for supervision, whereas prior work on medical abstraction requires sentence-level or note-level annotations that are harder to acquire at scale.

#### Case finding

Case finding can be framed as binary classification over a patient’s clinical documents from a given day. We use the same architecture as in Figure 6 and find it similarly effective. (The models are learned separately for case finding vs. abstraction. We conducted preliminary experiments on multi-task learning but did not find a significant difference in performance, as each task has abundant training data.)

Case finding poses a distinct self-supervision challenge. We can easily identify positive examples from the registry (patients with their diagnosis dates). However, it is less clear how to identify negative examples. We explore two self-supervision schemes. Initially, we randomly sample non-cancer patients and days from their medical history with pathology reports. This yields a classifier with good sensitivity (recall) but often incorrectly flags prediagnosis days for a patient with cancer, causing a high false-positive rate. To address this problem, we experiment with adding hard negative examples from patients with cancer by sampling days before diagnosis. The resulting classifier not only distinguishes patients with cancer from non-cancer patients but also identifies the time of initial diagnosis, as required for case finding. Together with abstraction, we can thus help accelerate cancer registry curation end-to-end.

## Data Availability

The EMR data for this study are not made publicly available due to privacy and compliance considerations established by the research protocol. Queries about these data should be directed to the corresponding authors indicated above. The PubMedBERT foundation model and its pretraining algorithm are detailed in Gu et al.^7^ PubMedBERT is made publicly available: https://aka.ms/pubmedbert. OncoBERT reflects a similar domain-specific pretraining approach as PubMedBERT but is trained on EMR data. While this model is not made available due to privacy and compliance considerations, the same approach can be used to train an analogous model at any site using the EMR data available. Additional source code supporting this study is made available at GitHub (https://github.com/microsoft/cancernlp) and has been archived at Zenodo.^25^
